# Supplementary material for: Forecasting admissions in psychiatric hospitals before and during Covid-19: a retrospective study with routine data
Source: Sci Rep. 2022 Sep 23;12:15912. doi: 10.1038/s41598-022-20190-y (PMC9508170; doi:10.1038/s41598-022-20190-y)
Supplement: Supplementary file 1 — Supplementary Information. [file 41598_2022_20190_MOESM1_ESM.pdf]

Table S1: Names and explanation of features

Feature	Explanation
Day of the week	The day of the week, i.e. Monday, Tuesday, etc.
Weekend	Monday to Friday vs. Saturday and Sunday
Public holiday	Days that are public holidays in the state of Hesse, Germany
School holiday	Days that are school holidays in the state of Hesse, Germany
Quarter	The quarter of the year, i.e. first, second, etc. (as factor)
Month	The month of the year, i.e. January, February, etc. (as factor)
Bridge day	A day between a public or school holiday and a weekend
23rd - 31 Dec.	The days between 23rd and 31st of December
Lag (14/35)	The number of admissions 14/35 days before the predicted day, because the number of admissions was not known yet on day 7/28 before
Fourier Series	The terms from a Fourier Series with weekly and yearly seasonal periods
Climate and weather features (at the day of prediction)	
Wind speed maximum	Maximum wind speed in meters per second
Wind speed mean	Mean wind speed in meters per second
Cloudiness	Mean cloudiness from 0 to 8
Air pressure	Mean air pressure in hectopascal
Precipitation depth	Depth of precipitation in millimeter
Precipitation type	Type of precipitation
Sun shine	Duration of sunshine in hours
Snow height	Height of snow in millimeter
Air temperature minimal surface	Minimal air temperature on the surface in Celsius
Air temperature mean surface	Mean air temperature on the surface in Celsius
Air temperature minimal 2m	Minimal air temperature in 2 meters height in Celsius
Air temperature mean 2m	Mean air temperature 2 meters height in Celsius
Humdity	Mean relative humidity in percent

Feature	Explanation
Google trend data (at the day of prediction)	
Google Trend "Depression"	Relative number of search queries in Hesse, Germany, for the German word: Depressionen
Google Trend "Sadness"	Relative number of search queries in Hesse, Germany, for the German word: Traurigkeit
Google Trend "Sad"	Relative number of search queries in Hesse, Germany, for the German word: Traurig
Google Trend "Suicide"	Relative number of search queries in Hesse, Germany, for the German word: Selbstmord
Google Trend "Mania"	Relative number of search queries in Hesse, Germany, for the German word: Manie
Google Trend "Fear"	Relative number of search queries in Hesse, Germany, for the German word: Angst
Google Trend "Panic"	Relative number of search queries in Hesse, Germany, for the German word: Panik
Google Trend "Dread"	Relative number of search queries in Hesse, Germany, for the German word: Furcht
Google Trend "Addiction"	Relative number of search queries in Hesse, Germany, for the German word: Sucht
Google Trend "Dependence"	Relative number of search queries in Hesse, Germany, for the German word: Abhängigkeit
Google Trend "Alcohol"	Relative number of search queries in Hesse, Germany, for the German word: Alkohol
Google Trend "Drugs"	Relative number of search queries in Hesse, Germany, for the German word: Drogen
Google Trend "Schizophrenia"	Relative number of search queries in Hesse, Germany, for the German word: Schizophrenie
Google Trend "Psychosis"	Relative number of search queries in Hesse, Germany, for the German word: Psychose
Google Trend "Hallucinations"	Relative number of search queries in Hesse, Germany, for the German word: Halluzinationen
